# A hypertension risk score for adults: a population-based cross-sectional study from the Dubai Household Survey 2019

**DOI:** 10.4178/epih.e2021064

**Published:** 2021-09-08

**Authors:** Ibrahim Mahmoud, Nabil Sulaiman, Amal Hussein, Heba Mamdouh, Wafa K. AL Nakhi, Hamid Y. Hussain, Gamal M. Ibrahim

**Affiliations:** 1 College of Medicine, University of Sharjah, Sharjah, United Arab Emirates; 2 Baker Heart and Diabetes Institute, Melbourne, Victoria, Australia; 3 Department of Data Analysis, Research and Studies, Dubai Health Authority, Dubai, United Arab Emirates; 4 Department of Family Health, High Institute of Public Health, Alexandria University, Alexandria, Egypt; 5 Department of Community Medicine, Mohammed Bin Rashid University of Medicine and Health Sciences, Dubai, United Arab Emirates

**Keywords:** Hypertension, Blood pressure, Cardiovascular diseases

## Abstract

**OBJECTIVES:**

The aim of this study was to develop a risk score model for predicting hypertension specific to the population of Dubai in the United Arab Emirates (UAE) to facilitate prevention and early intervention.

**METHODS:**

A retrospective analysis of data from the Dubai Household Health Survey 2019 was conducted. Demographic and physical parameters, as well as blood glucose levels, were included in the data. The risk factors for hypertension were identified using bivariate analysis. A risk score model was developed using the enter method, where all significant predictors of hypertension in bivariate analyses were entered in a single step with the primary outcome of hypertension status (yes/no). The model was validated internally by splitting the data into Emirati and non-Emirati populations.

**RESULTS:**

A total of 2,533 subjects were studied. The significant risk factors for hypertension identified were male sex, older age (≥40 years), education level, body mass index, diabetes mellitus, and dyslipidaemia. The model showed a high discrimination ability between individuals with and without hypertension, with an area under the curve of 0.77 (95% confidence interval [CI], 0.75 to 0.79), excellent sensitivity (81.0%; 95% CI, 71.9 to 88.2) and moderate specificity (56.0%; 95% CI, 45.7 to 65.9).

**CONCLUSIONS:**

The model developed by this study is simple, convenient, and based on readily available demographic and medical characteristics. This risk score model could support initial hypertension screening and provide an effective tool for targeted lifestyle counselling and prevention programs.

## INTRODUCTION

High blood pressure (hypertension) is a global public health concern that causes high morbidity and mortality and a substantial economic burden. According to the World Health Organization (WHO), over 1 billion people throughout the world have hypertension, which increases their risk of ischaemic heart disease, stroke, and kidney disease [1]. Hypertension is a major cause of premature death worldwide, and its annual treatment cost was estimated to be over US$24 billion in 2018 [2].

The WHO reported that the WHO African Region had the highest prevalence of hypertension (27%) of all regions evaluated [1]. A recent study among the Emirati population living in the northern region of the United Arab Emirates (UAE) found a higher prevalence of hypertension (31%) [3]. An even higher prevalence of hypertension (34%) was found among expatriate male workers from South Asia living in the eastern region of the UAE [4]. Furthermore, a high burden of undiagnosed hypertension was reported in the UAE [5].

Modifiable hypertension risk factors exist at a high rate in the UAE population. The UAE has the highest prevalence of overweight and obesity, diabetes, and dyslipidaemia in the world due to several factors, including rapid urbanization, which can lead to sedentary lifestyles [3,6,7].

Epidemiological studies suggest that targeting persons at high risk of developing hypertension or treating it at an early stage may delay its onset or lessen its consequences, respectively [8-10]. Several studies have attempted to develop risk score models for predicting the development of hypertension or the presence of hypertension based on readily obtainable demographic and medical characteristics [11-15]. Evidence has indicated that the Framingham Heart Study risk score improved the prediction of incident hypertension and facilitated the identification of individuals who were at high risk of developing hypertension [16]. However, those studies might not be applicable to the Dubai population due to differences in genetic backgrounds, lifestyle factors, and environmental factors. Environmental and lifestyle factors, including food, obesity, physical activity, cigarette smoking, alcohol intake, and environmental contaminants, are increasingly being shown to alter epigenetic mechanisms and patterns in populations [17].

To the best of our knowledge, no studies in the UAE or in Dubai have used risk scores to predict the development of hypertension. The aim of this study was to develop a simple and informative risk score model appropriate for the Dubai population using factors that can be readily obtained. This risk score model could support initial hypertension screening and provide an effective tool for targeted lifestyle counselling and prevention programs.

## MATERIALS AND METHODS

### Study design

This study accessed data from the Dubai Household Health Survey (DHHS) that was conducted in 2019. The DHHS is a population-based cross-sectional questionnaire that was designed to assess the health status of the Dubai population. The main domains of DHHS are health behaviours, non-communicable diseases, healthcare delivery, and health expenditures. Residents in Dubai (Emirati and non-Emirati) were recruited for the DHHS using a complex stratified clustered random sampling technique.

### Setting and participants

Dubai, with a population of around 3 million, is the second-largest city and Emirate in the UAE. Adults aged 18 and above, who account for roughly 80% of Dubai’s population, were the target population for the 2019 DHHS and were sampled using a cluster sampling method. The study sample available within the secondary data is limited to the number of respondents in the 2019 DHHS who received 3 successive measurements of systolic and diastolic blood pressure.

### Variables and measurements

The socio-demographic variables included in this study included age, sex, marital status, nationality, education, occupation and work status, and lifestyle habits, including smoking, physical activity status and alcohol consumption. Weight, height, and systolic and diastolic blood pressures were measured to obtain participants’ body mass index (BMI) and blood pressure status. Diabetes status was determined based on haemoglobin A1c (HbA1c) cut-off measures. Participants were divided by age into 2 categories, 18-39 years and ≥ 40 years old. Marital status was categorised as married, single and divorced/separated/widowed. Nationality was categorised into Emirati and non-Emirati; and educational level was categorised as below secondary, secondary, and tertiary. Physical activity status was categorised as active and not active, with active participants engaging in at least 150 minutes of moderate-intensity aerobic physical activity throughout the week, at least 75 minutes of vigorous-intensity aerobic physical activity throughout the week, or an equivalent combination of moderate-intensity and vigorous-intensity physical activity [18]. Participants were defined as current smokers if they reported any type of tobacco smoking. Alcohol consumption was defined as any alcohol usage during the month preceding the survey. A BMI of < 25.0 kg/m^2^ was considered to indicate normal weight, 25.0-29.9 kg/m^2^ was defined as overweight, and ≥ 30.0 kg/m^2^ was considered indicative of obesity. Hypertension was defined as self-reported high blood pressure in the medications section and/or a blood pressure of ≥ 140/90 mmHg [19] as measured during the survey. Diabetes status was determined using an HbA1c test. The cut-off values for the test were defined as follows: < 6.5% was considered non-diabetes and ≥ 6.5% indicated diabetes [20]. Participants with self-reported diabetes in the medications section were also considered to have diabetes.

Data were collected by trained research assistants provided with standardised guidelines. Blood pressure was measured and recorded 3 times. The average of all 3 measurements was considered the most accurate and was thus recorded. Blood pressure was measured at 2 points in time after the first measurement, with 10-minute intervals between the measurements.

### Statistical analysis

To describe the demographic and clinical characteristics of the population, frequencies (in percentages) for people with hypertension and those without hypertension were reported.

Bivariate analyses (chi-square) were conducted to identify the variables associated with hypertension. The non-statistically significant variables were eliminated. Statistical significance was set at p-value < 0.05.

Multiple binary logistic regression analyses were performed using the enter method, wherein all significant predictors of hypertension in the bivariate analyses were entered in a single step with the primary outcome of hypertension status (yes/no). The risk score for this study was calculated in 2 stages: First, a score was computed by multiplying the regression coefficients by 10 and rounding to the nearest integer for each significant variable in the multiple logistic regression analysis. Second, the risk score for an individual was calculated by adding the scores for each risk model variable. The estimated risk score was evaluated using a receiver operating characteristic (ROC) curve and the area under the curve (AUC) to derive a population cut-off based on optimising the sum of sensitivity and specificity. The data analysis was performed with IBM SPSS version 26 (IBM Corp., Armonk, NY, USA).

Reporting followed the STROBE (Strengthening the Reporting of Observational Studies in Epidemiology) statement for cross-sectional studies.

### Ethics statement

The Dubai Scientific Research Ethics Committee, Dubai Health Authority, approved this study (DSREC-GL03-2021). A signed informed consent form was obtained from all participants.

## RESULTS

**Figure 1.**
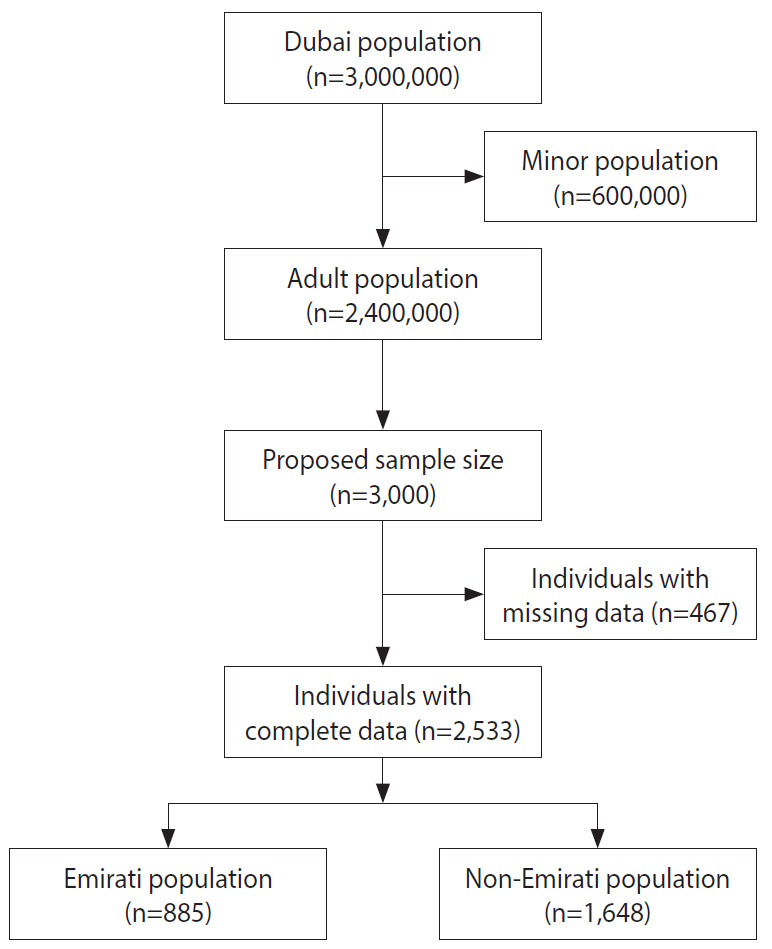
Flowchart of the study participants.

There were 3,000 eligible subjects who participated in the study, of whom 2,533 (84.4%) had complete blood pressure data (Figure 1). Of the 2,533 study participants, 1,503 (59.3%) were male. The mean age of the entire sample was 40.8± 14.3 years old. Table 1 shows the characteristics of the study participants stratified into Emirati and non-Emirati groups and based on their hypertension status (yes/no). Bivariate analyses revealed variables that were significantly associated with hypertension (p≤0.05).

Tobacco use and alcohol consumption status were not statistically significantly associated with the presence of hypertension (p>0.05); therefore, these were excluded from our final models. Physical activity status and marital status were also excluded from our final models after stepwise forward modelling adjustment. The variables included in the final logistic regression models were sex, age, educational level, BMI group, dyslipidaemia status, and diabetes status, with the primary outcome of hypertension status (yes/no). Three binary logistic regression models were developed in this study: a binary logistic regression model to explore the factors associated with hypertension on the Emirati cohort; another model for the non-Emirati cohort and a combined model for both the Emirati and non-Emirati cohorts (Table 2). Being male (odds ratio [OR], 2.51; 95% confidence interval [CI], 2.01 to 3.15), over 40 years old (OR, 1.95; 95% CI, 1.57 to 2.42), or obese (OR, 3.12; 95% CI, 2.39 to 4.04) and having dyslipidaemia (OR, 1.62; 95% CI, 1.27 to 2.06), or diabetes (OR, 2.93; 95% CI, 2.33 to 3.70) were significant positive predictors of hypertension. Secondary education (OR, 0.58; 95% CI, 0.44 to 0.75) and tertiary education (OR, 0.48; 95% CI, 0.37 to 0.62) were significant negative predictors of hypertension (Table 2).

### Model validation and performance

Internal validation of the model was performed on the 2019 DHHS data. The data were split into 2 cohorts: Emirati and non-Emirati. Three regression models were developed for the 2 cohorts and the combined data (Table 2). The regression beta coefficient values obtained from the Emirati and non-Emirati models were highly similar to those of the combined model.

**Figure 2.**
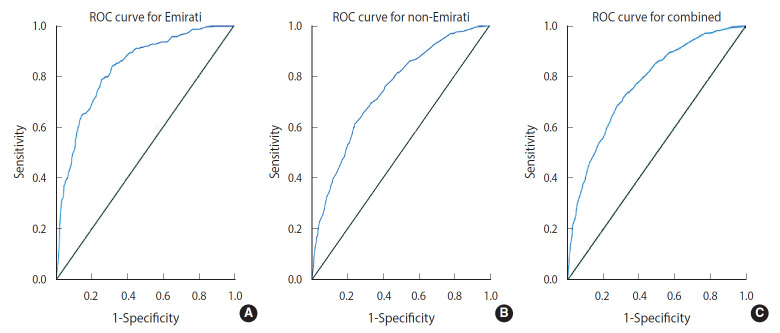
Receiver-operating characteristic (ROC) curves demonstrating the hypertension risk score model’s performance in predicting hypertension (A) Emirati, (B) non-Emirati, and (C) combined.

Table 2 also shows the performance characteristics of the 3 models. The Emirati model showed an excellent ability to discriminate between those with normal blood pressure and hypertension, with an AUC of 0.83 (Figure 2A), while the non-Emirati model showed an acceptable discrimination ability, with an AUC of 0.74 (Figure 2B), similar to the combined model, which had an AUC of 0.77 (Figure 2C). A cut-point of ≥ 25 was determined from the coordinates of the curve of each model in order to maximise the sum of sensitivity and specificity. The combined risk score showed an excellent sensitivity of 81% and a moderate specificity of 56% for predicting hypertension (Table 2).

## DISCUSSION

In the current study, a risk scoring algorithm to predict individuals in the UAE who are likely to develop hypertension was developed. We analytically assessed a set of factors that were identified in the literature as predictors for hypertension in order to develop a simple and convenient model. The study model indicated that obesity, diabetes, male sex, old age, and dyslipidaemia were strongly associated with an increased risk of hypertension, while tertiary education was inversely associated with hypertension. Our model showed a high capability of discriminating between individuals with and without hypertension, with high sensitivity and moderate to fair specificity. Our findings are consistent with those of several studies from the region and worldwide that have identified similar variables as predictors of hypertension [21-23].

The study identified a strong association between high educational attainment and a low risk of hypertension after controlling for possible confounders. Although the acquisition of education might not always translate into behavioural change, we propose that high educational attainment is often connected to individuals’ income, understanding of hypertension’s risk factors and consequences, and access to prevention methods. The effect of low educational attainment might continue even after the development of hypertension, as a study from Canada in patients with hypertension showed that those with low educational attainment were, in general, less likely to engage in lifestyle behaviours for blood pressure control [24,25].

Several studies have revealed that smoking and alcohol consumption are independent predictors of hypertension [21,26,27]. Interestingly, this study did not find a statistically significant association between hypertension and smoking or between hypertension and alcohol consumption in either Emirati or non-Emirati populations. We argue that these predictors might be under-reported in a conservative community like that of the UAE, particularly among females, as only 7 persons in the Emirati population in this study reported consuming alcohol. Furthermore, these factors might not be necessary for the development of hypertension, although they are important predictors in evaluating the risk of cardiovascular diseases [13].

Our combined model showed a high discrimination ability between individuals with and without hypertension, with an AUC of 0.77, which is similar to that in the Framingham Study model (0.78) [14], a Korean model (0.79) [12] and a Taiwanese model (0.73) [15]. Our Emirati model, however, showed a better discrimination, with an AUC of 0.83. Furthermore, the Emirati model explained 40.4% of the variability in predicting hypertension, while the non-Emirati model explained 22.0% of the variability and the combined model explained 26.0%. These differences in performance and explained variability between our models could be due to heterogeneity among non-Emirati respondents, who might have different predictors related to their ethnicities. In addition, the percentages of explained variability indicate that there are other influential predictors of hypertension, such as a family history of hypertension and dietary intake, that were not captured by the current study.

From a preventive medicine perspective, this scoring model can be used in Dubai by health professionals in clinical settings to provide targeted lifestyle counselling for people with normal blood pressure about their predicted risk of developing hypertension and discuss with them appropriate preventive measures. This study identified educational attainment as an important protective factor. Hypertension is a silent disease and is usually asymptomatic; thus, people with low educational attainment might not be fully aware of its risk factors and consequences. Clinicians should provide people with high predictable risk scores with appropriate preventive strategies, including health education and weight, diabetes, and dyslipidaemia control. Furthermore, the model can also be used as an initial screening tool to identify people at high risk of having undiagnosed hypertension.

The strength of this study was using 2 large, community-based, heterogeneous cohorts (Emirati and non-Emirati) to validate the prediction model, which showed a high capability of discriminating between those with and without hypertension. Moreover, this study reported the screening characteristics of the developed models, including sensitivity, specificity, positive predictive value, and negative predictive value, which to our knowledge were not reported in previously published studies [12,13,15].

There were some limitations to this study, including that temporality between the predictors and hypertension cannot be established due to its cross-sectional design. Furthermore, the study data were not specifically collected for risk modelling purposes; as a result, some important demographic predictors, such as a family history of hypertension and dietary intake, were not assessed in this study. In addition, the study is based on data from residents of Dubai, who might not be representative of the entire UAE population.

In conclusion, this study developed a risk score model that is unique and suitable to predict the risk of developing hypertension for Emirati and non-Emirati populations living in Dubai in the UAE. It offers a simple and convenient tool based on readily available demographic and medical characteristics that can easily be used in clinical settings. This risk score model could support initial hypertension screening and provide an effective tool for targeted lifestyle counselling and prevention programs. Clinicians should provide people with high risk scores with appropriate preventive strategies, including health education and weight, diabetes, and dyslipidaemia control. We recommend conducting a prospective study that would include all emirates in the UAE to ensure generalisability, with the collection of additional predictors of hypertension and the inclusion of measurements taken over time.

## Figures and Tables

**Table 1.** Characteristics of study subjects based on nationality and hypertension status

Characteristics		Hypertension status					
		Emirati			Non-Emirati		
		No	Yes	p-value	No	Yes	p-value
Sex							
	Female	357 (70.8)	147 (29.2)	0.011	434 (82.5)	92 (17.5)	<0.001
	Male	239 (62.7)	142 (37.3)		714 (63.6)	408 (36.4)	
Age (yr)							
	18-39	338 (88.7)	43 (11.3)	<0.001	794 (76.4)	245 (23.6)	<0.001
	≥40	258 (51.2)	246 (48.8)		354 (58.1)	255 (41.9)	
Body mass index							
	Normal	193 (82.3)	32 (17.7)	<0.001	466 (78.5)	128 (21.5)	<0.001
	Overweight	148 (67.9)	70 (32.1)		381 (63.8)	216 (36.2)	
	Obese	139 (50.2)	138 (49.8)		137 (49.8)	138 (50.2)	
Physical activity status							
	No	531 (65.9)	275 (34.1)	0.003	1,011 (69.2)	450 (30.8)	0.255
	Yes	65 (82.3)	14 (17.7)		137 (73.3)	50 (26.7)	
Education level							
	Below secondary	124 (44.3)	156 (55.7)	<0.001	188 (57.3)	140 (42.7)	<0.001
	Secondary	252 (76.6)	77 (23.4)		308 (68.1)	144 (31.9)	
	Tertiary	220 (79.7)	56 (20.3)		652 (75.1)	216 (24.9)	
Marital status							
	Single	187 (89.9)	21 (10.1)	<0.001	351 (80.0)	88 (20.0)	
	Married	340 (64.5)	187 (35.5)		765 (66.5)	385 (33.5)	<0.001
	Divorced/separated/widow	69 (46.0)	81 (54.0)		32 (54.2)	27 (45.8)	
Tobacco use status							
	No	493 (67.6)	236 (32.4)	0.699	927 (70.4)	390 (29.6)	0.200
	Yes	103 (66.0)	53 (34.0)		221 (66.8)	110 (33.2)	
Alcohol consumption status							
	No	588 (67.1)	288 (32.9)	0.296	899 (70.8)	371 (29.2)	0.060
	Yes	6 (85.7)	1 (14.3)		243 (65.7)	127 (34.3)	
Cholesterol status							
	No	435 (75.7)	140 (24.3)	<0.001	995 (71.6)	394 (28.4)	<0.001
	Yes	161 (51.9)	149 (48.1)		153 (59.1)	106 (40.9)	
Diabetes status				<0.001			
	No	509 (79.5)	131 (20.5)		1,007 (76.3)	312 (23.7)	<0.001
	Yes	87 (35.5)	158 (64.5)		141 (42.9)	188 (57.1)	

Values are presented as number (%).

**Table 2.** Binary logistic regression models for the Emirati cohort and non-Emirati cohort and their performance

Variables		Emirati cohort			Non-Emirati cohort			Combined cohort		
		β-coefficient	OR (95% CI)	Score	β-coefficient	OR (95% CI)	Score	β-coefficient	OR (95% CI)	Score
Sex										
	Female		1.00 (reference)	0		1.00 (reference)	0		1.00 (reference)	
	Male	0.676	1.97 (1.33, 2.92)	7	0.946	2.64 (1.96, 3.55)	9	0.922	2.51 (2.01, 3.15)	
	p-value		0.001			<0.001			<0.001	
Age (yr)										
	18-39		1.00 (reference)	0		1.00 (reference)	0		1.00 (reference)	
	≥40	0.743	3.14 (1.99, 5.00)	7	0.545	1.72 (1.33, 2.23)	7	0.666	1.95 (1.57, 2.42)	7
	p-value		<0.001			<0.001			<0.001	
Education level										
	Below secondary		1.00 (reference)	0		1.00 (reference)	0		1.00 (reference)	0
	Secondary	-0.416	0.45 (0.28, 0.72)	-4	-0.317	0.73 (0.53, 1.01)	0	-0.446	0.58 (0.44, 0.75)	-4
	p-value		0.001			0.057			0.002	
	Tertiary	-0.793	0.32 (0.19, 0.53)	-8	-0.576	0.56 (0.41, 0.77)	-6	-0.588	0.48 (0.37, 0.62)	-6
	p-value		<0.001			<0.001			<0.001	
Body mass index group										
	Normal		1.00 (reference)	0		1.00 (reference)	0		1.00 (reference)	0
	Overweight	0.177	1.19 (0.68, 2.08)	0	0.465	1.19 (0.68, 2.08)	5	0.380	1.46 (1.14, 1.88)	4
	p-value		0.535			0.001			0.003	
	Obese	1.113	3.12 (1.84, 5.27)	11	1.234	3.12 (1.84, 5.27)	12	1.137	3.12 (2.39, 4.04)	11
	p-value		<0.001			<0.001			<0.001	
Dyslipidaemia status										
	No		1.00 (reference)	0		1.00 (reference)	0		1.00 (reference)	0
	Yes	0.647	1.92 (1.30, 2.84)	6	0.520	1.92 (1.30, 2.84)	5	0.480	1.62 (1.27, 2.06)	5
	p-value		0.001			0.001			<0.001	
Diabetes status										
	No		1.00 (reference)	0		1.00 (reference)	0		1.00 (reference)	0
	Yes	1.131	3.16 (2.09, 4.79)	11	0.997	3.16 (2.09, 4.79)	10	1.077	2.93 (2.33, 3.70)	11
	p-value		<0.001			<0.001			<0.001	
Cut-off score				≥25			≥25			≥25
Model performance										
	AUC	0.83 (0.80, 0.87)			0.74 (0.72, 0.77)			0.77 (0.75, 0.79)		
	Sensitivity	85.0 (76.5, 91.4)			80.0 (70.8, 87.3)			81.0 (71.9, 88.2)		
	Specificity	67.0 (56.9, 76.1)			54.0 (43.7, 64.0)			56.0 (45.7, 65.9)		
	PPV	52.5 (45.2, 59.6)			42.7 (37.1, 48.5)			44.1 (38.3, 50.1)		
	NPV	91.3 (86.5, 94.4)			86.3 (80.4, 90.7)			87.3 (81.6, 91.4)		

CI, confidence interval; AUC, area under the curve; NPV, negative predictive value; PPV, positive predictive value.

## References

[1] World Health Organization https://www.who.int/news-room/fact-sheets/detail/hypertension.

[2] Constant AF, Geladari EV, Geladari CV, Andreadis EA (2016). Hypertension and cardiovascular disease.

[3] Mahmoud I, Sulaiman N (2019). Dyslipidaemia prevalence and associated risk factors in the United Arab Emirates: a population-based study. BMJ Open.

[4] Shah SM, Loney T, Sheek-Hussein M, El Sadig M, Al Dhaheri S, El Barazi I (2015). Hypertension prevalence, awareness, treatment, and control, in male South Asian immigrants in the United Arab Emirates: a cross-sectional study. BMC Cardiovasc Disord.

[5] Abdulle AM, Nagelkerke NJ, Abouchacra S, Pathan JY, Adem A, Obineche EN (2006). Under- treatment and under diagnosis of hypertension: a serious problem in the United Arab Emirates. BMC Cardiovasc Disord.

[6] Sulaiman N, Mahmoud I, Hussein A, Elbadawi S, Abusnana S, Zimmet P (2018). Diabetes risk score in the United Arab Emirates: a screening tool for the early detection of type 2 diabetes mellitus. BMJ Open Diabetes Res Care.

[7] Rajan PB (2018). The growing problem of obesity in the UAE. Academicus ISJ.

[8] Hinton TC, Adams ZH, Baker RP, Hope KA, Paton JF, Hart EC (2020). Investigation and treatment of high blood pressure in young people: too much medicine or appropriate risk reduction?. Hypertension.

[9] Carey RM, Muntner P, Bosworth HB, Whelton PK (2018). Prevention and control of hypertension: JACC Health Promotion Series. J Am Coll Cardiol.

[10] Saptharishi L, Soudarssanane M, Thiruselvakumar D, Navasakthi D, Mathanraj S, Karthigeyan M (2009). Community-based randomized controlled trial of non-pharmacological interventions in prevention and control of hypertension among young adults. Indian J Community Med.

[11] Paynter NP, Cook NR, Everett BM, Sesso HD, Buring JE, Ridker PM (2009). Prediction of incident hypertension risk in women with currently normal blood pressure. Am J Med.

[12] Lim NK, Son KH, Lee KS, Park HY, Cho MC (2013). Predicting the risk of incident hypertension in a Korean middle-aged population: Korean genome and epidemiology study. J Clin Hypertens (Greenwich).

[13] Kshirsagar AV, Chiu YL, Bomback AS, August PA, Viera AJ, Colindres RE (2010). A hypertension risk score for middle-aged and older adults. J Clin Hypertens (Greenwich).

[14] Parikh NI, Pencina MJ, Wang TJ, Benjamin EJ, Lanier KJ, Levy D (2008). A risk score for predicting near-term incidence of hypertension: the Framingham Heart Study. Ann Intern Med.

[15] Chien KL, Hsu HC, Su TC, Chang WT, Sung FC, Chen MF (2011). Prediction models for the risk of new-onset hypertension in ethnic Chinese in Taiwan. J Hum Hypertens.

[16] Kivimäki M, Batty GD, Singh-Manoux A, Ferrie JE, Tabak AG, Jokela M (2009). Validating the Framingham hypertension risk score: results from the Whitehall II study. Hypertension.

[17] Alegría-Torres JA, Baccarelli A, Bollati V (2011). Epigenetics and lifestyle. Epigenomics.

[18] World Health Organization (2020). Physical activity. https://www.who.int/news-room/fact-sheets/detail/physical-activity.

[19] European Society of Cardiology/European Society of Hypertension (2018). Arterial hypertension clinical practice guidelines. https://reference.medscape.com/viewarticle/902759.

[20] World Health Organization (2011). Use of glycated haemoglobin (HbA1c) in diagnosis of diabetes mellitus: abbreviated report of a WHO consultation. https://apps.who.int/iris/handle/10665/70523.

[21] Ibekwe R (2015). Modifiable risk factors of hypertension and socio-demographic profile in Oghara, Delta State; prevalence and correlates. Ann Med Health Sci Res.

[22] Kingue S, Ngoe CN, Menanga AP, Jingi AM, Noubiap JJ, Fesuh B (2015). Prevalence and risk factors of hypertension in urban areas of Cameroon: a nationwide population-based cross-sectional study. J Clin Hypertens (Greenwich).

[23] Kilpi F, Webber L, Musaigner A, Aitsi-Selmi A, Marsh T, Rtveladze K (2014). Alarming predictions for obesity and non-communicable diseases in the Middle East. Public Health Nutr.

[24] Pandit AU, Tang JW, Bailey SC, Davis TC, Bocchini MV, Persell SD (2009). Education, literacy, and health: mediating effects on hypertension knowledge and control. Patient Educ Couns.

[25] Gee ME, Bienek A, Campbell NR, Bancej CM, Robitaille C, Kaczorowski J (2012). Prevalence of, and barriers to, preventive lifestyle behaviors in hypertension (from a national survey of Canadians with hypertension). Am J Cardiol.

[26] Wu J, Li T, Song X, Sun W, Zhang Y, Liu Y (2018). Prevalence and distribution of hypertension and related risk factors in Jilin Province, China 2015: a cross-sectional study. BMJ Open.

[27] Khan RJ, Stewart CP, Christian P, Schulze KJ, Wu L, Leclerq SC (2013). A cross-sectional study of the prevalence and risk factors for hypertension in rural Nepali women. BMC Public Health.

